# An open-label trial of alsuma auto-injector for migraine

**DOI:** 10.1186/1129-2377-14-S1-P185

**Published:** 2013-02-21

**Authors:** E Ramos, SH Landy, SJ Tepper, T Wein, E Schweizer

**Affiliations:** 1 Pfizer Inc, USA; 2 Wesley Neurology and Headache Clinic, USA; 3 Cleveland Clinic, USA; 4 McGill University, Canada; 5 Paladin Consulting Group, USA

## Objective

To assess the ability of patients, during an acute migraine attack, to successfully self-inject a single dose of sumatriptan using the Alsuma Auto-Injector; and to evaluate the safety and tolerability of the Auto-Injector.

## Background

The Alsuma Auto-Injector is a single-use system for the rapid subcutaneous delivery of 6 mg of sumatriptan succinate in the acute management of migraine pain. The Auto-Injector was developed to address the clinical need for an easy to use and rapid to administer system that did not require any assembly.

## Methods

This was an open-label, Phase 3 trial conducted at 10 sites in the US. Male or female adults, ages 18 to 60 years old, were eligible for study entry if they met IHS criteria for migraine with or without aura, with at least 2 attacks per month, and if they reported use of subcutaneous injectable sumatriptan on at least 2 occasions within the previous 2 months. During the onset of a migraine attack of moderate-to-severe intensity, patients were asked to administer a 6 mg subcutaneous dose of sumatriptan using the Auto-Injector. Subjects returned to the study site within 72 hours of the migraine for the post-treatment assessment visit.

## Results

A total of 63 patients met entry criteria and received a dose of study medication. All self-administered the Alsuma Auto-Injector successfully. On the patient questionnaire, 100% of patients agreed that the written instructions for the Auto-Injector were clear and easy to follow, and that the Auto-Injector was easy to use (95%). A majority of patients agreed that they preferred the new Auto-Injector to the traditional one that they were using prior to study entry (65.1%). The most frequent AEs was injection site bruising, reported by 15.9% of patients, and rated in all instance as mild in intensity.

## Conclusion

The majority of injection-experienced patients reported the pre-assembled, single-use Alsuma Auto-Injector to be an easy to use, preferred treatment for an acute migraine attack. The study found the Auto-Injector to be safe and well-tolerated.

